# In search of a principled theory of the ‘value’ of knowledge

**DOI:** 10.1186/s40064-016-3205-2

**Published:** 2016-09-20

**Authors:** Cristiano Castelfranchi

**Affiliations:** ISTC-CNR & Uninettuno, Rome, Italy

## Abstract

A theory of the Value/Utility of information and knowledge (K) is not really there. This would require a theory of the centrality of Goals in minds, and of the role of K relative to Goals and their dynamics. K value is a notion relative to Goal value. Inf/K is precisely a resource, a means and the value of means depends on the value of their possible functions and uses. The claim of this paper is that Ks have a Value and Utility, they can be more or less ‘precious’; they have a cost and imply some risks; they can be not only useful but negative and dangerous. We also examine the ‘quality’ of this resource: its reliability; and its crucial role in goal processing: activating goals, abandoning, choosing, planning, formulating intentions, decide to act. ‘Relevance theory’, Information theory, Epistemic Utility theory, etc. are not enough for providing a theory of the Value/Utility of K. And also truthfulness is not ‘the’ Value of K. Even true information can be noxious for the subject.

*Knowledge itself is power* (Francis Bacon)

## Premise

*Knowledge has/is a ‘value’.* Does this obvious claim have a theory, which explains it and how/why? I’m not so sure. We will argue that such a theory:

- is not the ‘Relevance theory’ from Pragmatics;
- it is not the Information theory;
- or the Epistemic Utility theory;
- it is not the *truthfulness* that gives value to a knowledge item

In our frame knowledge and its processing is not the essence of mind; thus our claim is that *the value/aim of knowledge* (any doxastic representation)^1^*is not (just) a stronger Cognition, a knowledge increment.*

The value of knowledge *has to be derived from* (*the value of*) *its Goals*, that is, from its use and utility; and also from its necessity and reliability. A doxastic information, representation it is just a resource; it is ‘power’.

### What is mind for?

Doxastic/epistemic information (Knowledge) is not the center, the end, the sense, and the real nature of ‘Mind’. The center of gravity of mind are Goals, which—on the basis of knowledge—have to successfully drive our behavior. Mind is a system for teleological ‘controlling’ conduct on the basis of ‘representations’ and their manipulation (assumptions on the current state of the world, on the powers of the Agent, future-augmented reality-imagination & anticipation-, desired states,…); a system built to ‘solve problems’ by working on the representation of them (that is, ‘mentally’), by reasoning, planning and deciding.

### Our thesis

This distortion in the view of Cognition is the reason why a theory of the Value/Utility of information in term of knowledge is not really there; systematically grounded and developed. This would require (as we said) a view of the role that K has *in relation to Goals* and their dynamics, management. K *value* is a notion relative to Goals and their Value. Since Inf/K is a means, the value of means depends on the value of their possible goals/uses. We search for, acquire, buy, preserve, use, consume,.. exchange… this crucial ‘power’^2^ for realizing goals.

The thesis of this paper (a preliminary exploration, not a complete systematization) is that Ks are just a fundamental resource; just *means* for (potential) goals. Ks have a Value and Utility, they can be more or less ‘precious’; they have a cost and imply some risks; they can not only be useful but negative and dangerous,… In a sense, one should apply to K—in this goal-oriented perspective—a ‘economic’ frame.

Of course, it is true that in humans the acquisition and storage of K has become an end in itself (and even an ‘intrinsic motivation’ and a ‘value’) (section “Goal processing and the utility of beliefs”). Subjectively we do not necessary search for knowledge acquisition instrumentally to a foreseen ‘use’ of it. Nevertheless, the *function* of K is instrumental. In human evolution, psychology, and society *means becomes ends* (this holds not only for K, but for social image and relations, for money, for power, for conformity to norms, etc.).

## “Relevance”, “information” etc. are not enough

We will discuss here—in a rather finalized way just in view of our objective and claims—the limits of Relevance theory, of Information theory, and of other approaches, that in our view do not solve the problem of the ‘utility’ and ‘value’ of information items and knowledge.

### ‘Relevance’ is not so clear and is not enough

It is not our aim here to discuss or develop the theory of ‘Relevance’; which, on one side, it is strongly bounded to pragmatics and communication theory (which are not our topics here); on the other side, it is quite rich and complex and not so clearly defined and systematized. We will give here a reductive view of ‘Relevance’ just in order to introduce some important ‘distinguo’. We do not need just the notion of ‘Relevance’ but those of ‘Utility’ and its measure, of ‘pertinence, etc. And we do not just focus on communication or mere cognitive processing, but on cognition for motivated action.

We have two main criticisms towards the Relevance notion and theory.

First, this notion doesn’t have a convergent definition and use,^3^ and even in the different domains and approaches it is not very clear and rigorously defined; it is based on rather vague notions.

#### Not just communication and knowledge processing

Second, our claim is that Relevance theory (we will focus on the most important version, by Sperber and Wilson 1986) is just centered on communication and/or cognition. It lacks one of its fundamental pillars (action and goals). It is quite strong in its foundation on (explicit) communication theory; but it is weak as for its cognitive foundation, which is not reducible to communication requisites and effects, and to *merely epistemic functions* and need for Information.

Let’s us—for characterizing in a synthetic way Relevance theory-refer to the very authoritative synthesis by Wilson and Sperber (2003).

“Relevance theory may be seen as an attempt to work out in detail one of Grice’s central claims: that *an essential feature of most human communication*, both verbal and non-verbal, is the expression and recognition of intentions. According to *inferential model of communication*, a communicator provides evidence of her intention to convey a certain *meaning, which is inferred by the audience* on the basis of the evidence provided… The goal of *inferential pragmatics* is to explain how the hearer infers the speaker’s meaning on the basis of the evidence provided. … *The central claim of relevance theory* is that the expectations of relevance raised by an utterance are precise enough, and predictable enough, to guide the hearer towards the speaker’s meaning.”

We can see how Relevance and its *theory* are explicitly and very strongly related to (linguistic) communication and to Pragmatics, in particular to Grice’s view of it.

When they move to generalization as for “Relevance and Cognition” they (correctly but very vaguely) claim that:

“Intuitively, relevance is a potential property not only of utterances and other observable phenomena, but of *thoughts, memories and conclusions of inferences*. In relevance-theoretic terms, any external stimulus or internal representation *which provides an input to cognitive processes* may be relevant to an individual at some time…. the search for relevance is *a basic feature of human cognition*, which communicators may exploit”.

They try to “introduce the *basic cognitive notion of relevance* and the Cognitive Principle of Relevance”:

“*When is an input relevant?* Intuitively, an input (a sight, a sound, an utterance, a memory) is relevant to an individual when it *connects[?] with background information* he has available *to yield conclusions thatmatter[?] to him*: say, by answering a question he had in mind, *improving his knowledge* on a certain topic, settling a doubt, confirming a suspicion, or correcting a mistaken impression. In relevance-theoretic terms, an input is relevant to an individual when its processing in a context of available assumptions yields a POSITIVE COGNITIVE EFFECT” (even identified with “*true conclusions*”! see below).

This is for us a very biased view of when and why information “matters” for a cognitive agent.

*Communication*—it is true—just exploits a much more general and foundational feature of *cognition*: acquiring ‘relevant’ information. But ‘relevant’, important, for what? If is not just for communicating and understanding messages. Why given information “matters” for a guy if we put aside the understanding of the other’s messages? What defines its ‘Utility’?

A more general and foundational theory of Cognition, K, and its functions is clearly lacking here; a view not just centered on doxastic structures and processes (like inferences) or on “true conclusions”. Their examples are typically just oriented to *epistemic* goals and functions. And the notion of “positive cognitive effect” (which should clarify the issue) is quite general and vague.^4^

“A *positive cognitive effect* is a *worthwhile difference to the individual’s representation of the world*—a true conclusion, for example. False conclusions are not worth having. They are cognitive effects, but not positive ones^5^ … An *efficient cognitive system* is one which tends to pick out genuinely relevant inputs, *yielding genuinely true conclusions*.”^6^

In other words, those strictly “cognitive” processing and effects/results/products (inferences, answers, arguments for, detection of contradictions,…) are *just a sub*-*case*, a restricted view of K, Inf inputs ‘uses’, utility, that is, of the goals they are useful for, and in particular a special kind of goals that are ‘functions’ (section “Goals and their value”). K and its processing is “for” something, and this makes a given piece of K more or less relevant or better ‘useful for’ a given (cognitive) function. But there are much larger set of non-epistemic functions and of explicit objectives and uses of epistemic inputs.

Given our different objective and our distancing from ‘Relevance’ as central notion, let us do not use for our main object the term of ‘Relevance’; this for two reasons:

- It is too strongly related to such noble tradition, to linguistics pragmatics, and to epistemic processes;
- We want a more general, basic, pragmatic (*action related*) term: like ‘Utility’, ‘Value’; related to practical (activity theory) notions similarly to other concepts like: ‘means’, ‘instrumental’, ‘useful’, ‘result’, ‘costs’, ‘risks’,….

Thus, we will sketch here a general and basic theory of Inf or Knowledge *Utility* and *Value*, as a crucial ground eventually also for Relevance theory (but this is not our aim here) and as a crucial ground in general for understanding human cognition.

### Not just ‘information’

Let’s shortly present the important contribution of Luciano Floridi on informativeness and Relevance, as grounded on probability and information theory.^7^

Floridi clearly enounces the problem, as: “When is information relevant? How can we analyze the concept of information *that interests*^8^*the agent?”*

He moves from classical Relevance Theory (“query”, “answer”…) and proposes: a probabilistic revision of it; a counterfactual revision; a meta-informational revision. With strong arguments and proposals. He synthesizes also disadvantages of Relevance theory; like:

- no account of relevant misinformation;
- no distinction between *informativeness* and *pertinence*^9^
- no distinction of *degrees* of relevance and hence of *epistemic utility*…

And his conclusion is that:

“Agents require a constant flow and a high level of processing of relevant information in order to interact *successfully***[?]** among them and *with the environment in which they are embedded*. Standard theories of information are silent on the nature of relevant semantic information. A subjectivist interpretation can account satisfactorily for several important applications and interpretations of the concept of relevant information. The interpretation provides the missing foundation for a general theory of relevance.”

**Fig. 1:**
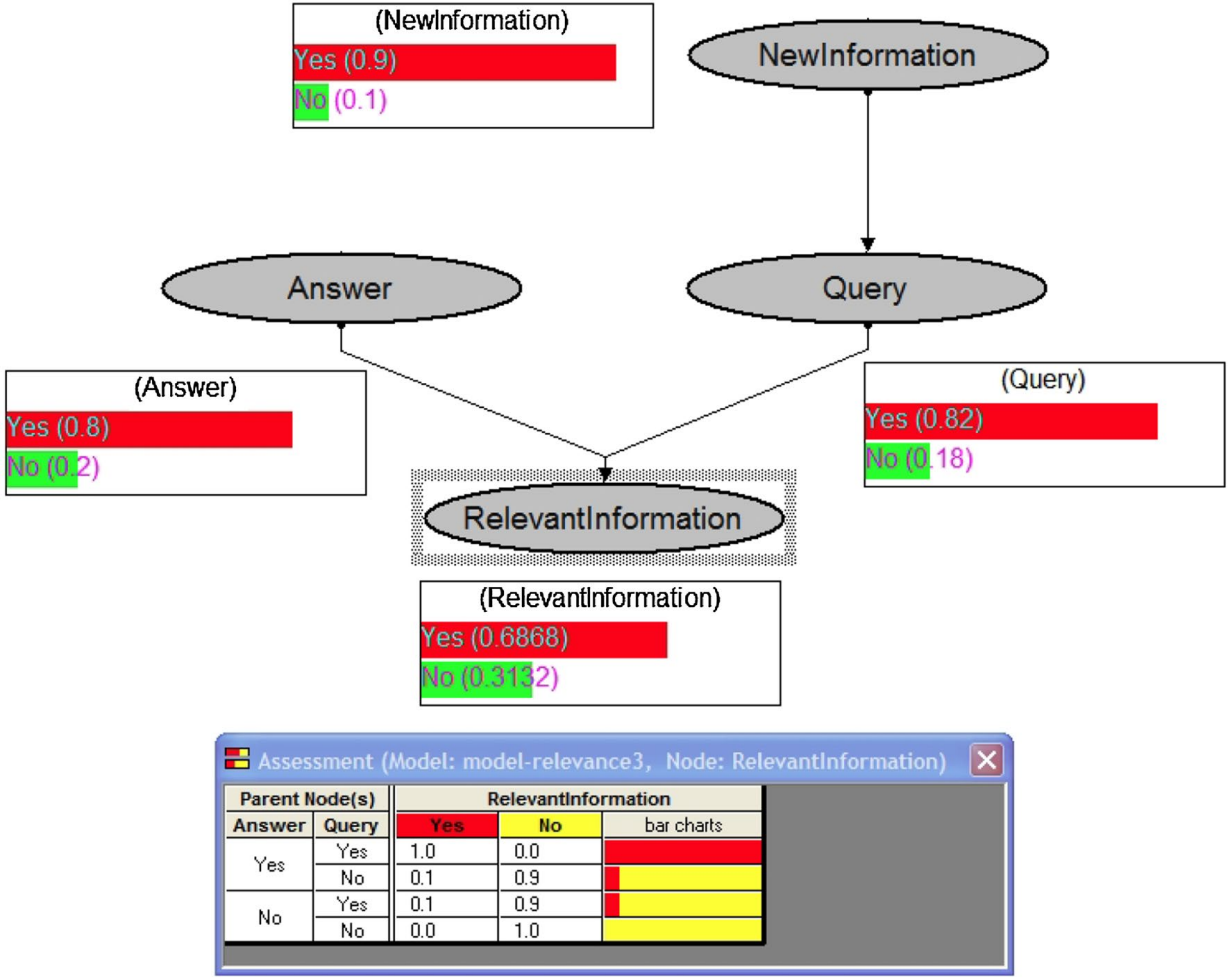
Relevant Information (from Floridi 2007)

This is true and relevant, however—in our view—it would be also required an explicit theory of why information is needed “*in order to* interact *successfully*” with the environment; that is, to act and to achieve goals. In his much more advanced model, Floridi however does not abandon the strictly “cognitive” view of Inf relevance and of its “*epistemic utility”.* The basic notions of the process model still are “question” “answer” etc. (see Fig. 1). It is not really explained what does it mean that a given input “matters” for the agent. His view of cognition seems putting aside an explicit/systematic theory of the motivational aspects and of the action regulation.

### Not just ‘information’ quantity

In general, in our perspective, “Information theory” cannot solve the problem of the *Utility* of the information items.

The mathematical theory of information is *based on probability theory and statistics*, and it also provides a *measure* of information, with several quantities of information (Shannon, Kolmogorov,..)

So, one might claim (within this frame) that:

- The more informative (in this sense) a given input, Inf item, the greater its Value

For us this is quite reductive and wrong.

It is possible that:

- A *less informative* data/input be much more relevant and *useful*for my specific issue and Goal

### Epistemic *utility* theory

Epistemic utility theory (Pettigrew 2010)—despite its name—deals with a quite different problem.

“How should *rational believers* pursue the *aim of truth*? Epistemic utility theorists have argued that the framework of decision theory can explain what it means for rational belief to have *the aim of approximating the truth*.” They follow Bayesianism and other theories of partial beliefs in modeling an agent’s epistemic state at a given time t by a belief function bt, which takes each proposition A about which the agent has an opinion and returns a real number bt(A) such that 0 bt(A).

“We take bt(A) to measure the agent’s degree of belief in A at time t.”^10^

“One of the central projects of formal epistemology concerns the formulation and justification of *epistemic norms*. Epistemic utility theory has so far furnished us with a number of arguments for *some of the central norms governing partial beliefs”*.

So the aim of this strong approach is the formulation of *norms for a rational*^11^*believing and truth approximation*; and notice that the ‘utility’ of an epistemic representation is just identified with the “truth” or better with: *the aim of approximating the truth.*

### Not just ‘economics’ of information

The ‘Economics’ of information typically focus on rather different problems.

#### Perfect or asymmetric information

The dominating part of economic theory is grounded on the assumption of “perfect information” in the economic subjects/actors. The most advanced and accepted model of the general economic ‘equilibrium’ in a market economics is based on such hypothesis that the subjects operating in the market are perfectly and equally informed. Only on such a basis, the market economy produces the optimal results (in Pareto’s sense) without any public intervention, as postulated by liberal ideology. These are the condition for a good working of the “invisible hand”.

This paradigm is considered too ‘normative’, idealized, from the new “economics of Information” for modeling a lot of real economic problems and dynamics, where the information accessible from the various subjects is imperfect; both partial and frequently wrong (opinions not truth). 2001 Nobel price to Stiglitz, G. Akerlof e M. Spence was given precisely for this revolutionary theory of “asymmetric information”. This asymmetry and different distribution of information is crucial for explaining for example the dynamics between a major and his agent, and their contract; or the problem of financial markets, or of credit, which is intrinsically connected with problems of getting the complete and correct information. And so on.

#### Epistemic utility

A very important approach is that on the “Expected utility *value* of a given information” Value of information (or epistemic utility) has been used in philosophy of science to model how much a truth seeker would be willing to pay for information (obtained, e.g., by running an experiment) prior to making a decision. Specifically, according to the standard view (Fallis 2000; Levi 1962), an agent has to make a decision, i.e., he has to choose one action among A_1,…, A_n. The value of a certain information (e.g., the value of running a given experiment) is identified with the difference between the highest value of utility the agent is expected to obtain in the actual situation and the highest value of utility the agent is expected to obtain after having obtained the information (e.g., after having run the experiment). In other words, the expected utility *value* of given information specifies *how much the information would improve the agent’s decision*.^12^ Moreover, in Fallis’ work a model is presented “that assigns different epistemic utilities directly to *different degrees of belief* that a truth seeker might have in a true hypothesis”; this is very close to the role we give to the degree of certainty grounded on specific sources and supports.

This theory is definitely interesting for the explicit instrumental relation put between information and utility maximization (the economic unifying and unique ‘goal’), and also it captures some crucial aspects of our frame. However, it is a bit restricted since it cannot give us a typology and an explanation of *how* and *why* that Inf increases our achievement probability and value (one should have a model of our different goals, of their processing conditions, and of the multiple relationships between beliefs and action).^13^ More in general, it captures only the role of Inf for the *decision making*, while—as we saw—there are various roles of K in relation to goals, which give value to K acquisition; for example the value of K for possible future goals (section “K quality”).

Moreover, this model can just give us a notion of the ‘subjective’ utility of that information, not of its ‘objective’ utility (section “Other distinctions”) for the subject and his achievement and profit.

A more recent and growing domain in economics (more management and business oriented) is the “infonomics”: focused on the central role of information as business ‘capital’, ‘asset’ (Moody and Walsh 1999), and the best strategies for selecting, acquiring, and exploiting precious data in front of the revolution of digitalization of the market relations (suppliers, clients, distribution,..), and with the explosion of Big Data, etc. (Laney 2012). These are important studies (with a correct *instrumental* view of ‘data’), but without any cognitive foundation or interest.

## Where is ‘pertinence’ theory?

As we said the notion of ‘Relevance’ has also mixed up and obscured another crucial notion: that of ‘Pertinence’,^14^ which, on the contrary, definitely deserves its own theory.

Information, data, and thus beliefs and knowledge are ‘about’ something; they inform specifically *ON* something, not on everything or arbitrarily. The obvious claim is that:

- Only ‘pertinent’ information on my issue can be useful for me (and be ‘relevant’).

If I get some information (even new and surprising) on a completely different and independent problem and domain, this doesn’t help me (for my given Goal G’); it has no value relative to it (it can be useful for new goals and activate new goals of mine).

But on the other side, it is not *sufficient* for being useful and having value that given information be ‘pertinent’ for that issue, be ‘about’ my objects and topic.

- Not all the information ‘about’ my current object (Obj) is useful for my goals on that object.

However, there is a basic problem before and for exploring this issue: *What does it means that given information is ‘pertinent’ for a given Obj and ‘about’ that Obj?*

Let us—in a cognitive frame—define this notion in a quite simple but direct way.

Mind works *with* and *on* ‘mental *representations’* (representations OF something!). Any representation has a ‘content’, an ‘object’. The objects of a ‘propositional representation’ are its Predicate and Arguments (it informs about them and their relation); the objects of a sensory-motor representation (an image, a perception) are its perceptual parts/components and their relations. The representation is “about” its objects (see Appendix 1).

Given a Goal G’ and its Objs, our claim is that *another representation (doxastic or motivational) is ‘pertinent’ for G’ (but not necessarily useful) IFF its objects partially overlap with G’ objects.*

However, these are the ‘directly’ pertinent informing data; but there are also ‘indirectly’ pertinent ones; which just ‘indirectly’ are ‘about’ the Obj.

- If from a given data or goal (R’) about Obj’’ I can *infer, derive* another data/goal (R”) on Obj’ (the Obj of my starting goal or belief) R’ becomes ‘indirectly’ pertinent, indirectly ‘about’ my Obj’.

Thus,

- Only a representation (doxastic or motivational) *directly or indirectly about* the contents of my Goal can be useful (and relevant) for me.

But, as we said, a lot of information that is ‘about’ can be absolutely irrelevant, useless, and mere noise.

### Network-based Pertinence

Reasonably, there is also another crucial factor for determining and predicting if a given K/D/belief is informing, is pertinent about X. It is the structure of the network of (episodic or generic) Ks ‘about’. Our representations—in particular the doxastic ones (data, beliefs,..)—are not stored as files or lists; they are *organized* in a specific way. Let us simplify by using the more clear and schematic ‘propositional’ format. The proposition (content of a possible belief) P’ is represented in terms of a given predicate (Pred) with its argument(s) (arg). For example B1: Pred (arg1 arg2); B1: Eat (John, bred). The organization of the data/Ks is *around* its arguments. We put around arg1 (John) all the beliefs or data we have directly about it, that is, mentioning it as an argument; and the same for all the data about arg2. If the predicate is not a simple single-argument ‘property’ but is a ‘relation (like ‘to eat’) this proposition (belief,..) connects the two (or more) nodes, and these representations and Ks, builds a Network.

**Fig. 2:**
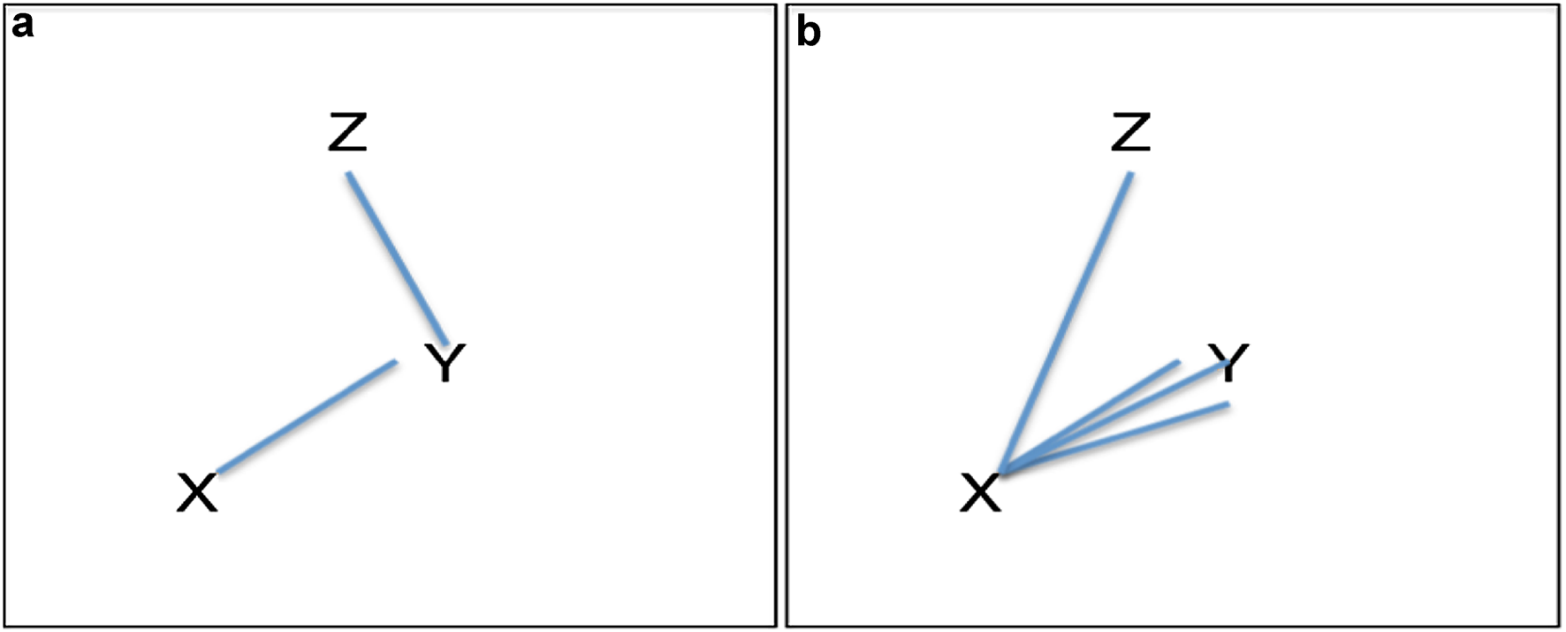
Our claim is that *the more connected Y with X, the more probable that an Inf (data) about Y be informative also about X, pertinent for having K about X*

Such network determines relation of direct vs. indirect connections, and of proximity; and also degrees of connection. For example, given the network in Fig. 2a, X is clearly closer to, more strongly and directly connected with Y than with Z. However, not only the ‘steps’ matters, but also the number of connections, the topology of the net. For example in Fig, 2b X is more connected with Y then with Z.

However, in our view this relation is not so trivial: *Is necessarily informative about X any K about/on Y if Y is strongly connected with X?* Not necessarily; not all the Ks about Y are pertinent for X. John has a dog, this dog yesterday has meet another dog in a garden; is that pertinent and informative about John? Non necessarily. But of course—in our view—even less informative about X is a belief about an entity not connected at all with X; a dog in another continent and ignored by John.

We remain on our claim: *to be pertinent for X, to give information about X, a K not directly about X must potentially be the basis, must give the opportunity of inferring something on X*. The relation with the network structure and its dimensions (proximity, number of connections) is this one: *the strongly related, connected Y to X, the more probable that a D/K on Y be informative (has something to say, to derive) about X*. If Johns’ dog gets fleas this probably has consequences for John, and we can infer, expect something about him; if a dog met in the garden has fleas this is for sure less pertinent and relevant about John.

So, the structure of connection between nodes is important, but the pertinence and possible informativeness about a node depends on possible inferences.

Another network structure is obviously crucial. In fact it is not just a matter of *topology* (proximity, etc.), it is also a matter of the semantics of the specific link. A very crucial link is the ISA relation: the link between a Class and its sub-classes and members (individual instances). Around the Class nodes we have the ‘generalized’ K (what we know about not a specific dog or car or action of eating but about dogs, cars, eating,..); this generalized information is there precisely in order to be inferred/instantiated on the members. So, if we (come to) know that X belongs to the Class C’, we automatically (come to) know a lot of things ‘about’ X.

Similarly, our K can be organized and assembled in ‘domains’ (like “sport”, “politics”, “art”,…). If my Obj of interest is within a given ‘domain’, probably the Inf within the same domain can be ‘indirectly’ pertinent for Obj. Or better, the probability that an Inf item in that domain be pertinent (and relevant) are greater than the probability of an Inf item of an independent/unconnected ‘domain’.

## Goals and their value

Since we will derive the V of K from the value of goals, we should first to rough out the theory of goals and of the bases of their Value. We will just remind this theory (which is quite rich and complex) in its very basic features, by just reminding the basic properties of goals that are necessary for this theory of K Value. However, we have, first of all to introduce a crucial distinction within two kinds of scientific teleological, finalistic perspectives and notions (and thus two possible meanings of “goal”).

A very important distinction—that we cannot extensively explain here—is between

- ‘Goals’ in psychological sense, as the mental anticipatory representation of a state of the world, moving and guiding—as a control device—the action which is “goal-driven or directed”); vs.
- ‘Goals’ in functional sense (we will use the term of ‘function’); that is, the result, outcome of a given feature or behavior which feedbacks on the entity and reproduces that feature/behavior, and thus itself. That ‘result’ is no longer a mere ‘effect’ but it is the ‘reason’ for the existence of that feature/behavior, which become “goal-oriented”, finalized, adaptive/useful for.

As for the theory of ‘utility’ of Inf items, of K, we will mainly focus here on explicit psychological goals, but not only on that: we will also examine a bit some pseudo-goals and functions, like K integration and support; or processing and selecting goal on the basis of epistemic inputs; or the ‘functions’ of our goal of curiosity, and of acquiring and just storing K.

### Goals^15^

A goal is *not a special kind of representation, different from a doxastic representation*; it is instead a mental representation with a specific ‘use’, ‘function’, ‘role’, ‘application’. Beliefs (etc.) and goals are just one and the same kind of representations, simply employed in two different ways. In fact, it has to be possible for such representations to match, in order to characterize the state in which an agent believes his/her goal to be satisfied. Beliefs and goals are two different possible ‘attitudes’ on the representations. For example, the same visual image can be used in a given circumstance as a belief about the current state of the world, and in another case as representing a state of the world to be achieved, i.e. a goal.

A goal is *not necessarily pursued*; a representation does not acquire the status of a goal only if and when it is being pursued as an ‘objective’.^16^ It is a goal (i.e., it plays the role of a goal) also in other stages of the control cycle; this is in fact the model of modern cognitive notion of ‘goal’ and of goal-directed behavior. Only in some cases, I have to ‘actively’ pursue my goals. When the realization is (believed to be) possible and depending on me, being up to me, then, either I subjectively “try” (the result is not subjectively sure) or I intentionally act for realizing it and confidently expect the desired outcome.^17^

In sum, a goal is a goal even before or without being pursued: satisfaction or happiness are due to goal-realization, and dissatisfaction or suffering are due to goal frustration, but not necessarily to our *active* successes or failures; we are sad because our mother has died, or happy because she gave us a kiss, even if we did not ask for it, nor do or expected anything from her.

There are different kinds of mental goals: *end goals* vs. *instrumental goals* (*means*) and their hierarchical structure; *plans*; *intentions*; *motivating and non*-*motivating goals*; *projects*; *expectations*,.. Let’s just focus on a difference that is relevant for the theory of goal Value: the difference between ‘felt’ and ‘non-felt’ goals.

#### Kinds of goals

“Goal” is *not a synonym of “desire”*. We do not accept to use as the basic, starting notion for possible motivated/goal-driven behaviors, the notion of “desires” (like in Bratman’s view, like in BDI logics, like in several philosophical and psychological uses). Desires are not at all the unique or general starting point of the motivational process. We have different kinds of goals, and some of them have nothing *desirable* in se’, but they just are instrumental, useful, or even oppressive or disagreeable duties.

Desires are just one kind and one possible origin of goals. They are ‘endogenous’ goals. When realized, they give pleasure, whereas not all goals provide us with a hedonistic experience once achieved (e.g., turning off the light can be an important goal, but doing so does not give us any particular pleasure).

This is important for a theory of decision and choice: in fact, *choices* are not only between ‘desires’ or attractive, desirable possible outcomes; we chose between desires, needs, practical things, duties,…; between harms or costs or threats; between plans, means, projects, programs,… not necessarily desirable in se’. Let’s go a bit more deeply on felt goals.

#### ‘Desire’ and ‘Needs’ as felt goals

‘Desire’ *in strict sense* of “desiring” something^18^; a specific mental state/activity and feeling. This desire is necessarily ‘felt’ (implying sensations), while not all the goals are “felt”; even not all the motivating goals are necessarily affectively charged and pleasant and attractive (at least in principle, in a general theory of purposive behavior). ‘Being desiring’ (“star desiderando”) in strict sense means the anticipated imagination of the ‘desirable’, pleasant, goal-state. The goal is represented in sensory-motor code and thus gives to the subject sensations (the flavor, the contact, the emotion,…) of the real object when realized. The subject is actually *imagining* these sensations and gets some ‘hallucination’, some anticipatory pleasure (foretaste). This can even imply not only the activation of ‘somatic markers’ (the central neural trace of previous somatic experiences), but the actual activation of the body, sending sensations: ex. salivation, erection, etc.

We do not “feel” all kinds of goals; we just have, formulate, an “intention”, a “project”, a “purpose”, a “plan”, etc. Instead, we “feel” desires and needs, and this is not by accident; they imply active sensory-motor representations (either “imagined”, evoked from memory, or current proprioceptive signals).

Not all our goals (even terminal ones, motivating us) entail real felt ‘pleasure’ when realized.^19^

#### Needs

Another *felt* kind of goals are “needs”. A felt need is due to a bodily sensation (current stimulus or evoked sensation).^20^

- Needs—differently from ‘desires’—are under the effect of negative, painful sensations, activating an avoidance-goal. Also for this reason we experience them as ‘necessities’, constraining us, ‘obliging’ us to do or not to do something.
- We also conceptualize and conceive a “need” as a *necessary* means, as the only possible solution for our goal: not only if I have O (what I need (for G))^21^ I can realize G, but if I do not have O I cannot and will not realize G. This gives to the general notion of “need” a sense of necessity, no choice, which—in the felt needs—is reinforced by the unpleasant sensation pushing us.

#### Emotions and felt value of goals

Although ‘motivation’ is not necessarily related to ‘emotion’ (see Appendix 2), the relation is important and also can affect not only goal activation but also goal Value.

There are goals with a felt (affective) *value* not only because of bodily sensation of pleasure or pain like in needs or desires, but because they are connected with emotions; either because activated by an emotional reaction or because they evoke an affective experience (like in Damasio’s “somatic markers”; Damasio 1994). These goals have a *value* also due to what we feel and to its intensity. This holds for example for avoidance goals frequently associated to fear, worries,.. or for goals related to important moral or esthetic or ideal “values” of us, or for goals within affective relationships, or for forms of hostility related to envy or to resentment, and so on.

### The origin of goal value

Since we have more than one goal and several goals can be active in the same time and context, and be in conflict (incompatible), this is why we have to ‘choose’, to have ‘preferences’. But this implies that goals have to have a subjective ‘value’, that is, a value perceived or assigned by the decision-maker: a quantitative dimension enabling the decision maker to give them some ordering, by making them preferable or not to other goals.^22^ Our issue here is the following one*: Where does the value of a goal come from?*

There are *two different sources and kinds of value,* which may interact (either in synergy or in conflict) with each other.

- The “calculated” value (a), based on means-end relations (Pascal’s “reasons of the Reason”);
- The just “felt” and intuitive value (b) (Pascal’s “reasons of the heart” that the Reason cannot understand, since they are not arguable, reasoned, but just felt).
  - One kind of value is “calculated”, based on means-end relations, on the examination of possible advantages (“pros”; achieved higher-goals) and disadvantages (“cons”; frustrated goals); the value of a given goal is derived from its forecasted consequences: from the number and values of the realized higher-goals, and the number and values of the frustrated ones (“costs” in broad sense). This value is ‘inherited’ from the goal hierarchy, from the ends to their means.^23^
    Clearly, this value is based on our beliefs and in particular our predictions (although automatic, memorized) and it is in fact ‘justifiable’: I have my explainable ‘reasons’ for choosing a given alternative; I have decided to go by train and not by car, because so and so. This is in fact the domain of persuasive argumentation (while marketing and advertising mainly work on the second kind of value: affective unconscious associations).
  - The other kind of value is not reasoned upon or calculated, but just felt and intuitive, and not really justifiable. It is due to the (frequently unconscious) activation of affective responses conditioned to analogous experiences and to the ‘intensity’ of such sensation, feeling elicited by a given anticipatory representation or memory. How intense and unpleasant is the sensation due to a felt need, to a negative disturbing stimulus from our body (hunger, thirst, sleepiness, tiredness,..) such that “I need”, “I feel that I need…”? How intense the pleasant sensation of a foretasting, of imagining what I desire and expect? How intense the negative emotion (fear, disgust, …) elicited by an Avoidance goal (a representation of something that I’m pushed to avoid, to prevent)? This gives the degree of value.

#### Pascal’s conflict

Given these two possible “values” of the goals and forms of appraisal (not necessarily present at the same time) they may converge or conflict (Miceli and Castelfranchi 1989; see also section “Satisfactory not optimizing”): one and the same goal can be both very “attractive” and very “useful”; or it may be high in “utility” but not attractive at all; or very attractive but extremely costly and irrational.

We not only have two independent, parallel, and competitive systems for regulating our behavior, for making a given goal prevail: one unconscious, automatic, fast, evocation based, affective, etc.; the other based on reasoning and deliberation, slow, etc. (like in “dual system” theories, nowadays very popular: e.g., Sloman 1996; Sun 2002; Kahneman 2003). These systems strictly interact with each other; more precisely, the affective, evocative system enters the space of deliberation, introduces new dimensions on goals (value) and beliefs (strength), and alters the process and result of our reasoned decisions.^24^

#### ‘Pleasure’ and goal value

This also means that in our model pleasure is not *“the”* goal of our activity, its final motivation; and the same holds for feeling pleasure (or avoiding feeling pain). “Pleasure”—as a specific and qualitative subjective experience, sensation (not as an empty *tautological* label for ‘goal satisfaction’)—normally is not a goal for us: it is not what we intend to realize/achieve while acting, what move us for performing that behavior. Of course, feeling pleasure or avoiding pain *might* become real goals and intentionally drive our actions: that is basically the mindset of the true hedonist, who acts for pleasure and not for whatever practical consequence his/her action accomplishes. But typically looking for pleasure and avoiding pain are not a unique final goal of ours (a monarchic view of mind and motivation): rather, they act as ‘signals’ for learning, and they help us learning, among other things, how to generate and evaluate goals.

In our view, pleasure is more related to the notion of ‘reward’, of ‘reinforcement’ and learning. Pleasure as an internal reward plays two fundamental roles: it attaches some value to some achieved state, which is important when the system can have more than one of such states, possibly in competition with each other; it signals that a given outcome (perhaps accidental) ‘deserves’ to be pursued, is good, has to become a goal (that state, not the pleasure per sé). In this view, pleasure is a learning device for goal creation/discovery and for evaluation. It seems very useful in a system endowed with a ‘generative’ goal mechanism, and which needs different kinds of evaluation, more or less intuitive, fast, based on experience or on biological/inherited ‘preferences’, and not just on reasoning (with its limits, biases, and slowness).

## The value of knowledge and its origin

The Value of K strictly depends on its relation with Goals; in particular, on three crucial roles K plays:

- *Technical datum*; means for pursuing and achieving G (to know how, to know existent conditions, a crucial information like the combination of the safe, etc.).
- Beliefs are the *support* of our preferences and action: if I loose my expectation that that result is ‘possible’ (my ‘hope’) I will drop out my goal; the same for my belief that G1 is better than G2 (beliefs on the Value of the Goal, or better beliefs that *give* Value to the Goal) (see section “Goal processing and the utility of beliefs”).
- Beliefs are frustrating or gratifying; that is, they *are* the failure or realization of our Goal. Achieving a goal (in a cybernetic model) just means that the epistemic representation and the motivational one do match; achieving a goal just means to *believe* that the goal is achieved.^25^

### Deriving the K value

As just said, a piece of information (data or belief) is ‘relevant’ or better ‘useful’ only relative to some Goal: either it represents/is the ‘frustration’ or the ‘satisfaction’ of the Goal; or it is a necessary condition for a given action for achieving the Goal. Let’s focus on the second case.

- The Knowledge Value (KV) is due to *the value/importance of the goal G’* (*G’V*)

This is a bit too simple; the theory of Value presupposes the theory of ‘evaluation’. Given that we do not have only one goal but open sets of goals; and we may have a set of currently ‘active’ goals, and a set of possible or future goals, we have to consider the Utility of a given Inf or K in relation not just a single (and actually pursued or taken into account) goal, but *in relation to a given Set of goals (Sg)* considered by the subject in its evaluation. Thus, *the value of K is due to the value/importance of the goal*-*set Sg it is useful for, and their value.*

The evaluation (attribution of ‘Value’) to a given entity Obj in fact is usually multi-dimensional; there is not just one parameter, one *standard*^26^ or criteria (in other terms one current or possible use and goal) relative to whom we appraise the correspondence and utility of Obj. This implies also that there is a sum of partial values of Obj. It can be ‘good’ in relation to goal or Value G1 & G2 & G3, and thus be more precious of an Obj just useful/good for G2. However, it can also be very good for G1 but frustrating, harming G2. Thus the Value of Obj is the value of G1 minus the Value of the frustrated goal: it is partially good and partially bad.

Consider for example (section “The costs and risks of K” ii) when the subject decides to revise a lot of her previous knowledge, to made a ‘revolution’ of her view about Obj. The new Inf—although very costly from that point of view of abandoning previous certainties, of restructuring a K world, of revising a lot—is so convincing, authoritative, not rejectable that she decides to accept it. Clearly enough here there is a conflict, and then an *ambivalent evaluation* (value) of K’: on the one side, a negative value (the cost of revising, destroying other sources and trusts), on the other side, a positive value; which presupposes another goal (explicit or implicit (pseudo-goal)): not only to have integrated and supported beliefs, but also the goal of having the *best ones, the most credible and supported ones, from the best sources.* This goal (which gives the positive value/utility to K’) is clearly prevailing on the goal of preserving previous knowledge, of not working too much for revision.

b. The degree of “contribution”

Second, K Value is due to t*he degree of “contribution” of that piece of K* to the choice, plan, action, or G achievement. A given K/D can contribute more or less: it can increase more or less the probability to achieve that goal.

- The more precious/crucial is K/D for achieving G, and the greater the value of G, the greater the value of K/D for the subject.

In other words, Data are not equally *precious*; and this predicts:

- The probability to memorize, to preserve, or to forget them;
- The probability to search for them (the value of the epistemic goal of acquiring them).^27^

#### Useful or necessary?

However, also the idea of the ‘contribution’ of the K item is too vague and incomplete; one should in particular be more precise on a fact we mentioned: that D/K—as any other ‘tool’—can be *useful but not necessary*; that is, it might have alternatives. *The many the alternatives to that K’ the lower its KV.*

In order to achieve G’, I need D1 *OR* D2 (not D1 & D2). Suppose that either D1 or D2 are sufficient for realizing G1: if I have/access D1 I can achieve G1; but also if I get/have D2 I realize G1. Neither D1 nor D2 is needful. Which is the value of D1 or D2? It is the Value of G1 simply divided? (Except they have a different probability to bring to G1).

This also makes the KV context dependent. In fact, K replaceability or necessity can be related to a given context C: a given K can be accessible or not in a given C but not in another; and make more or less precious its alternative. In context, C’ to achieve that G it is necessary to have/use K’; but not in C”.^28^

This KV has interesting contextual dynamics. Consider this example:

- In order to achieve G we need three data, we have to know: D2 & D2 & D3. The value/importance of the Goal is in some sense divided for the number of necessary data: no one of them is *sufficient* (just the set), all of them are *necessary*.
- In order to achieve G we already have/acquired D1 and D2 and we just search/expect for D3, and if we obtaining D3 we realize our goal G. At that point, in that situation all the value of the goal is concentrated on D3: now, it is necessary and sufficient for G.

(So, there is a strange dynamics of the value: the value, importance of “acquiring” versus the value of not forgetting, of preserving, memorizing).^29^

#### Necessity and adjustable goals

An important kind of ‘necessity’ of a given information (and in general of a given resource) is when we have goals that can be ‘partially’ realized.

There are goals that are “Yes/No” “All/Nothing”: either we achieve it or not (for example, to get a PhD qualification). However, there are other kinds of goals that can be achieved just in part: either because they are ‘gradable’, they refer to a quantity^30^; or because they are composed of sub-outcomes, and one can achieve a sub-set of the global goal. The problem is if that part is satisfactory for realizing (enough) that goal. (For example, our goal was to have a nice trip and vacation: this part was excellent, this was very good, but this event/experience was bad).

Now, a given resource or tool can be necessary for the full and complete achievement of the goal, but not indispensable, essential: even without it the goal is OK. For example, I have to prepare a given special food: without the meet and the potatoes the food is not there at all; without fire, cooker, and oil I could not cook it; without salt it is not a good food; however, it would be better to also put some oregano inside, and I do not have it. However, it is not essential; the food is OK, and is almost perfect.

The same holds for Inf Items; some Inf Items might be useful for a full realization of my goal, but some of them are good if there but not indispensable.

#### Belief strength and contribution

The degree of contribution of a given Inf item (belief) for achieving my goal by deciding and by doing something (thus it ‘utility value’) is also due to its degree of credibility, certainty (section “K quality”). Stronger beliefs contribute to a given choice and decision to do, more than the doubtful ones. Suppose that for a given choice I need six beliefs, but all of them are not very sure; perhaps I will suspend my choice or renounce to my goal. Suppose now that only two beliefs are quite doubtful while the others are quite sure; it is possible that this is for me enough for taking that decision. Thus, the role/effect of the various beliefs in this decision was not equivalent; the decision is more ‘due’ to the stronger beliefs than to the weakest ones (that might have deter me from). So *the more certain the belief the more impact it has on the choice or decision to act* (section “Goal processing and the utility of beliefs”).

### Other distinctions

#### Subjective or objective value?

Moreover, there is also another crucial and basic aspect of ‘value’ on which one should be more precise: its ‘subjective’ or ‘objective’ character.

There is a *‘subjective utility’* of a given resource (in this case of a given K/D), that is the perceived/evaluated usefulness by the agent, which determines how much it is desired and searched for (the value of the doxastic goal to know it). But, there also is an *‘objective value’*^31^ of K’ for G’ of Agent’. Not necessarily our evaluation are correct and rational or realistic; not necessarily we really understand what we would need and the utility of things/actions. For example, our ‘interest’ (what would be better for us and our goals, or—vice versa—contrary to our good) frequently does not coincide with our desires and objectives/decisions. We can do not understand what is ‘in our interest’, and do not realize that we do not understand it: we believe/feel that we know it.

The principles presented here should both apply to the subjective KV estimated by the subject and to the objective value in the perspective of an ideal observer. However, notice that the Set of goals that is used for evaluating G’ by the Subject can be and usually is different from the Set of goals taken into account by the external observer for his ‘objective’ evaluation of the Value of that K for the subject.

Of course, also for K *‘Utility’* (also the subjective one) *is different from ‘Pleasure’*. That a given D/K gives us some ‘pleasure’ can just be an aspect or kind of utility (since feel pleasure can be one of our goals). Also a disagreeable D/K can be very useful. For example, if I have to demolish a lot of my previous integrated K about Q, to revise and work a lot, this might be very unpleasant (and I could even resist to K in order do not do that) but can be very useful. Saying nothing of the case when subjectively to come to know K is very bad and undesirable (I would prefer do not know that), but in reality, objectively, this is very useful for me (other goals of mine).

#### Potential Value

There is a V of a K in a given situation, context, for a given and specified Goal (as for achieving G1 the value, utility of K’ is tot). However, it is also important to realize that:

- Human beings have a generative and open set of Goals; we generate and activate new Goals;
- A given resource or tool is not just for one and unique plan and goal; resources can be multipurpose and multiuse; and the same holds for Ks.

Given that, there is a just ‘potential’ Value of a given K item or domain: for possible future goals and uses; not for an active and specific goal to choose and pursue.

Now, the larger the perceived/expected *amplitude*, the imagined *set of goal families* K’ is useful for (its ‘polyvalence’), and the more important those goals families the higher K’ utility; although quite vague and just potential. Why money has become the dominant objective in human activity? Because it is a means for everything and thus becomes an end in itself (like K).

#### Class-goals

Not only there are vague sets of events but even conceptually defined classes. As we saw, doxastic representations, for example, propositional beliefs, can be specific and episodic (about a given event and object) or ‘generic’, that is, about a Class. The same applies to Goals; we my have an event goal or a class/generic goal: the goal that here and now Black be happy is different from the Goal to make it happy always when possible.

*If a given resource or K is useful for a class*-*Goal* it is useful for any possible instantiation of it; thus *it is more precious than a K useful only for one shot*.

#### Presupposed value

We even acquire knowledge that we do not need now and that we believe that is completely useless for us, stupid, boring, etc. “Who care! Why I have to learn that!”

However, first, there is in fact some current goal determining the importance/value of having/using such a K (the approval of professor; the vote on the report card; etc.). Second, I can trust/believe that it will be probably useful for future goals of mine; I trust them (parents, professors) that it is useful for me, has value, to learn that. Third, this is exactly a case of ‘tutorial’ role in K management as a social ‘institution’. We construct the ‘institution’ of K that people *has to have* in this community, for our identity and tradition, or for playing possible future roles. You do not understand that and do not have these goals now, since you follow your current desires not your future ‘interest’. But K as a collective institution also obliges and prescribes some learning (school) independently on your current use/utility, and current interests. In sum, this is the ‘normative’ way we build the K capital of our society; what “count as” instruction and knowledge.^32^

### K quality

Our doxastic representations have a ‘quality’: a subjective ‘certainty’. We are more or less “sure”, “convinced” that P.

In our model the degree of certainty, the strength of a belief, depends on its origin, on the sources; on the basis of two main principles:

- The more reliable, trustworthy (competent, honest) the source the more sure its information and my believing in it.^33^
- The many the convergent sources the more sure I feel (Castelfranchi 1997).

The *degree of certainty* should affect the KV: for example, I should ‘pay’ a given K from a very trustworthy source (or a K with a given degree of certainty) more than from a not so reliable source or a doubtful K. The degree of certainty (*quality* of K) has a important value since we actually bet and risk on that; we decide to spend our resources and actions on the ground of what we believe; so our trust in what we believe exposes ourselves to failure, harms, …

K is a resource that changes its value on the basis of its origin, of the brand, of ‘seller or producer’; or because the source is/gives a value in itself (like in dogmatic knowledge) or because the quality of its products has been proved superior (previous experience, reputation, marketing,..).

In sum, the Value of K is affected by its degree of certainty: the more sure, grounded, the more precious it is.

The value of uncertain and doubtful information is clearly inferior.

### The epistemic integration value of K items

There is also another utility and value of Inf items (data, candidate ‘beliefs’), not directly relative to specific ‘motives’, neither as frustrating/satisfying, nor as tool for realizing the goal. There is an ‘importance’ or ‘value’ of a given data or beliefs *just in relation to knowledge organization, integration, mutual consistency and support*. In a sense this importance or value is due to the ‘pseudo-goal’ of having robust, integrated, knowledge. To have coherent and justified knowledge is a crucial ‘function’ of our cognitive system (for example Thagard 2000).

Any process of knowledge acquisition, generation or elaboration does not only generate some output knowledge item; it also generates a trace of its origin, and ‘relation’ that supports and integrates such a new item. It generates at the same time knowledge structure (*network*) or ‘relational knowledge’: *Reasons* to believe.

This is true not only for inferences, but in general. Knowledge items remain related to their source: “I saw that p”; “I think that p, because…” “The TV said that p”, etc. Thus, there is a special relation between the Belief that p, and the Belief “I saw that p” or “the TV said that p” or “Since Q then P”.

Consequences of this ‘trace’ and relation theory are the following ones:

- Items are integrated in cognitive nets: you cannot eliminate or insert a new item of K, without dealing with its supports and relations. This is the well-studied problem of Belief revision and updating: changes are never merely local.
- Part of the difference between various ‘mental attitudes’ (like: belief, knowledge, opinion, prediction, etc.) is to be recalled to the “story” and the support of the proposition: its ‘Reasons’.
- We maintain in our mind both: Reasons to believe, and Reasons to Do

We talk about ‘support’ relations, because cognitive items hold thanks to such relations.

Now, this cognitive ‘need’ of *reason* to believe, of *support* and *integration* of K, this structure gives to K items different ‘role’ and ‘importance’ or ‘value’. *Not all items are equally important in a given domain, context, or episode* (independently on their degree of certainty and on goals). There are K items more ‘central’, ‘important’, ‘crucial’ while other items are just ‘marginal’, just ‘details’. This depends on their network role: is this piece of K supporting and explaining many other Ks of that episode or domain? What earthquake would happen if this K would result wrong? How much belief-revision work we should do? Or this information is quite irrelevant, it doesn’t support or explain nothing, and we can cheaply abandon (drop, revise, forget) it?

In other words, K items have different *value* and *utility* in relation to the need for coherence, support, and *argumentation* within our beliefs. It is more probable (ad reasonable) that we forget or put aside marginal details (that is, facts that are not important for understanding the whole, that do not explain the other facts and the global event) then central facts. It is more probable that we resist more to revise and abandon crucial, central, important items than irrelevant, marginal one.

In sum, K has *a peculiar form of Utility and Value*—its importance—just relative to doxastic (not motivational) aspects: how much it is integrated in the K nets and how many K items does it support or is supported by, and how central is in the topology of the Net?

### Goal processing and the utility of beliefs^34^

Mind is based on a *belief*-*goal bridge*: the real backbone of cognition.

- Beliefs support goals (beliefs as reasons for goals)
- Beliefs determine goal value
- Beliefs determine goal processing and dynamics
- Beliefs determine goal species

But this also gives Value to beliefs, make them more or less important from another point of view (different from the previous ones).

*Beliefs support* goals, by acting as *reasons* for them. A cognitive agent is an agent who grounds his actions on his beliefs: or better, he acts on the basis of what he wants and prefers (goals), but he wants and prefers on the basis of what he believes. In a cognitive agent, goals should be supported and justified by *reasons* (not necessarily unbiased and “rational”). We *activate, maintain, decide about, prefer, plan for, and pursue,* goals that are grounded on pertinent beliefs (supported by other beliefs, etc.). Goal-processing is belief-based.

Let’s summarize the role of different types of beliefs in filtering the goals and in regulating their transition step by step, from their activation to the action execution (for extended discussion, see Castelfranchi and Paglieri 2007).

Goal processing has in fact *different phases*, like: goal *activation*; *choice* between various active and competing goals; goal *planning and formulation of an Intention* to do something; *execution* of the planned action. Now, all these phases require specific information input and exploit specific beliefs. So, a given belief can be the condition for the *activation* of a given desire (for example, I see that there is an open ice-cream shop in front of me); or a belief is the condition for *abandoning* a given goal (the believe that it is impossible or the belief that between two active goals there is a contradiction, a conflict, and we have to choose between them); or a condition for *choosing* G’ and not G2 (for example, I believe that this dress that I personally like less that the other will be more appreciated by John and I want to like to John); or a condition for *formulating the intention* and the planned action (for example, to believe that such action has those results and that I’m able to perform it); or a condition for the action *execution* (for example, the information that it is the right time). Failing these belief tests would stop the processing of a goal (e.g., putting it in a sort of mental waiting room, until the agent beliefs will allow reactivating them), or may even eliminate certain goals; on the contrary, success of these tests will make a certain goal persist until the choice, the planning, the execution of the pertinent actions.^35^

Now, given that a given belief B’ is responsible for the acceptance of that G as ‘to be possibly pursued’ (up to me), or of the choice of that G as better than its competitors, or as a possible ‘intention’ of mine (I’m able and in condition) etc. it acquires a special ‘supporting’ Value. If B’ is revised, I have to drop that G; and this may be a cost, a waste; or I don’t want to renounce to G for its importance and attractiveness. This crucial role of B’ may entail unconscious ‘refusal’ to revise it^36^ and even self-deception in order do not renounce to my G or do not frustrated it.

### To know just for to know

We have (for evolutionary and cultural reasons) the final Goal of acquiring knowledge, the intrinsic motivation of curiosity. Thus knowledge and its acquisition can be/have a Value per sé.

However, first, curiosity is not for everything, omni-directional (at least, it is focused on certain domains: our ‘interests’, in order to restrict an infinite search). But this means that we are ‘interested’ in that, that is, we have *the goal to acquire K in that domain*, on that topic.

Second; it is true: we have the final goal to ‘cumulate’ and ‘store’ knowledge also without a clear immediate use of; like for money, or like ants for food. K it is a ‘good’ per sé. However, *not all the founded Inf has the same value*. Like for money it is different to put away 1000§ or 10§. *How do we evaluate the value of a storable K item, which is not immediately to be used for a given active goal G’?*

The generic goal to acquire knowledge is insufficient for explaining that. We plausibly evaluate in an approximate and intuitive way the *presumable* Value of that new information either on the basis of our ‘interest’ in the domain/topic it is Pertinent for (an area we know that we will have goals of specific uses), or in term of potential possible uses/goals (section “Other distinctions”). Like when I decide to buy a dress that I do not need just now for a specific ceremony (or I do not want it will be used only for that) and I evaluate the probability of other future occasions and good uses of it: “this dress is better; I’m sure that I can use it for a lot of other occasions and roles”.

So, even for the INF/K acquired just for the goal of acquiring K, there are additional Values/Utilities relative to not just to ‘know’, but to possible uses, interests.

## Other utility-related features and value effects

### Value losing

#### Redundancy

Of course an Inf, a piece of K, in order to have value for X should first of all not be already possessed by X, already known. If it just is a duplicate it has no value for X. To have value an Inf must be new or different, that is ‘informative’.

Nevertheless, the issue is a bit more complex. In fact, sometimes it can be useful and desired also to get the repetition of a previous (even believed) information. An already possessed K can have a utility as ‘confirmation’ of the previous one. A non-marginal function; especially in relation to the strength/quality of K, and of the credibility of its source (section “The epistemic integration value of K items“). To be true and more precise, in this case in fact a ‘new’ K is there, and it is this new K that makes useful its ‘object’; the is a meta-K that K’ is repeated (repeatable) and confirmed. For example, if X already believes/knows that P, but search for and get the information that “also the source S” supports P, or that the original source S’ repeats and insists “that P”, this is useful for X (it can reinforce P certainty). However, notice that the relevant and new information is about the sources of P.

#### K consumption and expiration date

Strangely enough some K has a “consumption”: once used is no longer useful; and thus it looses its value. Other Ks can be used several times, and/or for different goals. This looks strange since K is a tool, a resource that doesn’t wear out with use; or better it just waste a part of its possible V: its ‘novelty’ and ‘surprise’ (Lorini and Castelfranchi 2007).

However, even without ‘wearing out’ there might be different reasons for the ‘consumption’ of a given K item. For example, if John already answered to Mary, and said that news, I cannot do the same; I cannot ‘inform’ Mary about, as a new item. Or if we are in the Prisoner Dilemma and what matters is who confesses first, the confession of the second one is useless.

Moreover, that K items might have an ‘expiration date’; that is, being useful just in that moment and context and no longer. For example: (a) the number of the RSA necessary for Internet banking, but changing every minute; or a password with a deadline. (b) A precious information but for a rapidly evolving phenomenon, like the rise at stock exchanges, or weather. Or like a question for an examination/test: I give a wrong answer, it is no longer useful that later I learn the right one.

Either there is a deadline in the ‘use’ of that item: it is true but no longer required; or there is a deadline in the K: it is no longer ‘true’.

### The costs and risks of K

A piece of K also has *costs*.

(i) Acquisition costs:^37^

How much one has to invest (or have invested) in order to have K; in terms of time, effort, material resources (money,..), relations, sufferance,….

Obviously, the cost should be acceptable relative to KV current or possible or for multiple uses.

Nice examples of costs for acquiring K, are: to study; to pay an investigator; an intelligence service; reasoning and calculating; to memorize and store and to try to retrieve, etc.

It is also very relevant the relationship between the costs of K acquisition and the risks.

#### Satisfactory not optimizing

Because of these acquisition costs it not always the best to acquire additional knowledge for deciding. Simon’s theory of bounded and limited rationality is not only a matter on impossibility of computation (we cannot—for computational limits—acquire all the pertinent and even relevant Inf); it is also a matter of economics, of costs versus value. We cannot wait and defer our decisions by continuing to expect or search for additional relevant Inf; we have to decide. We have to renounce to an optimal or better solution, and accept a “satisfying” one. In fact, the cost of additional search, control, reasoning,.. and the risks of deferring are at a certain point higher than the possible increment of the achieved value (choice). The additional K is not convenient, is even negative (loosing opportunities): costs and risks are higher than advantages (see also section “Not just ‘economics’ of information” and Fallis 2000).

Complete information is frequently useless and unusable (for time, noise, costs,..). Suppose that Google would provide us *all* the information related to our question, however nobody spend times for reading more than the first 5-10 retrieved items or the first two pages!

(ii) Acceptance and revision, integration costs

Since our K—at least the explicit one and in the same domain—has to be coherent and justified (section “The epistemic integration value of K items”), how much work have I to do in order to include a new information? What I have to drop and abandon and how much this is difficult? How much the revised beliefs were important for my goals or for their role in the integration and support of the other Ks, or for my trust in its source? How demanding is the revision not just of that belief but also of the network integrated with it?

Beliefs revision is not a local operation and requires a lot of work also because the revision feedbacks also on other Ks and on the reliability of the sources. Have I to discredit a source? Can I do that? How much revision and integration work would this require.

The *integration****value*** (section “The epistemic integration value of K items”) of a given K is proportional to the ***cost*** of its revision; and the estimated Value of a new entry should be definitely superior to the revision costs due to its acceptance, and to the integration role/value of the dropped items.

(iii) Costs in use

There are not only acquisition cost; a piece of K, a data can be more or less expensive in its ‘use’, since it can be more or less difficult for retrieval, for derivation, for reasoning about. There are ‘difficult’ or ‘simple ‘notions’, and memory retrieval, or reasoning or verifications.

(iv) Risks *for* K and *of* K

Obviously there are serious risks when we do NOT have a given crucial K/Inf (see note 37); but there are also serious risks in or for having a given K.

- There are risks in the path for *acquiring* a given Inf—since we have to expose ourselves—, or in having such information (for example, a dangerous ‘secret’). But also risks in investing and working for having it. Or a risk while trusting a given source and Inf and ‘deciding’ to believe it.
- There also are risks in *using* a given K/D; for example, to believe that a guy is trustworthy and rely on him exposes us to serious risks of failure, of betrayal, etc. Or to count on given information exposes us to errors, wrong reasoning, wrong decisions, etc. (see below about ‘negative K’)

Let’s go a bit deeply in the possible ‘negative’ value of K.

## Useless and harmful Inf

Not all Inf is precious or at least useful; some Inf (even K in strict sense: “true”; see below) can be useless (for a given agent in a given context) and even noxious. It would be better do not getting or having it. K/D/Inf dangerousness has different faces and kinds:

- In a strict sense: to know that P, is harm or can bring harm to X:
  - Usually this holds when P is false, the Inf is wrong;^38^
  - Or when the K that P induces sufferance (“It would be better do not know it!”); however here it is matter of priority/importance of the goals: the truth or the sufferance?;
  - Or K is even true but misleading for X; or induces wrong inferences and beliefs; or induces X to a worse choice.

An Inf item (a belief) can be harmful for example because the agent builds—on such a basis—a wrong plan, and doesn’t achieve her goal. For example, X plans to go with her car to the town center for going to theater; she believes that at that time the town center be accessible by car (but this is wrong), so she goes with the car but cannot access the center, and misses the spectacle since arrives too late. If she would have known the truth, she would have made another more effective plan.

Analogously, an Inf Item can determine the preference and choice between two goals. I have to decide If to take or not a medicine and a friend of mine says that that drug gives nausea; then I decide do not take the drug. Notice that the belief of my friend can be wrong (a wrong association); however, also a true Inf can be in this case noxious by inducing me to avoid an useful drug (for my goal of not experiencing nausea it is useful/good; for my goal and interest of cure it is noxious/bad). This is why medical doctors frequently do not explain to patients the so-called ‘collateral effects’ of drugs; because they know that a lot of patients would not take them (See below on dangerous true K).

- Or to believe P is harmful since it is *not convenient*:
  - The cost was too high for its utility; not worth it
  - It was in fact useless but costly searching for it or reasoning about it;
  - It could be cognitively biasing, deviating attention, overloading, or confounding.

Thus, there is a Negative Utility of Inf/K, which is not just its acquisition, elaboration, conservation cost, but also risks and harms it produces.

The agent can be even aware, can believe, that a given Inf Item is dangerous.

A K item cannot just being *objectively* (from an observer’s point of view) harmful for an agent and her goal achievement, but also *subjectively*. That is, the Ag may believe that to get that Inf is/would be bad for her, by creating more uncertainty or confusion, or by activating automatic or affective reactions deviating for her correct decision and behavior.

An agent can obviously believe that a certain Inf item is necessary for her (and search for it), or believe that it would be irrelevant, superfluous, not useful (she has already enough Inf, she has already decided or achieve her goal); but she can also believe that receiving that Inf be noxious. For example, a person that knows to be too anxious and that anxiety would paralyze her decisions or action, or a person that is phobic of certain conditions, has the goal to *avoid* to receive Inf that might activate that reaction.

### ‘Knowledge’ in strict sense and the dangers of truth

We are sorry for Mill (1823) but there can absolutely be ‘noxious’ K; not all K is by definition good for humans, as he claims. This is even clearer if the parameter is not our “good” but our subjective “happiness” (not the same!).

Relative to our “happiness” how many times we sincerely say “I would have absolutely preferred do not know that! I suffer a lot and cannot do anything at all!”. Or even, what I now know obliges me to decisions that waste my life and I wouldn’t like to take. I would prefer to live in ignorance or deception.

We would like to avoid a deep discussion about what is “knowledge” in strict sense (true epistemic representations). As we said we use K as a broad notion (like used in AI, cognitive science) covering different doxastic and epistemic representation: Information, data candidate to be assumed or not as belief or K, beliefs with different degree of certainty, presuppositions, as-if assumptions, hypothesis, expectations,….).

Our view of “truth” is radically pragmatic (related to the theory of action and goals): that a given assumption, presupposition, explicit belief, subjective ‘knowledge’^39^ is *efficacy*; that is, that an action grounded on such a belief (on the world, on me, etc.) and relying on such represented ‘state on world’ be successful (goal achievement). A “true” knowledge is—in this radically pragmatistic sense—intrinsically ‘useful’ and of Value, since it is the basis of the action and of its success. “To be true” is a value and standard, and gives value. Nevertheless, consider that there is not only one goal in our mind and in our ‘evaluations’. For example, to “use/rely on” that assumption and to “verify” it, are not one and the same goal. The belief in the truth of a given K item, implies the possible action and potential *goal* of “verification”, an epistemic action aimed at looking at, and seeing if, and verifying or falsifying if P. Also relatively to that goal (motivating that action: that our assumptions be verified) that K is more or less ‘useful’, not only in relation to the ‘practical’ goal of achieving G’. And the results, success, could be different.^40^

Also for this radically pragmatistic notion of K, it follows that any K (as such) has value, since we have the goal of the truth and that what we believe be true^41^; but *it doesn’t follow that such a INF item since it is true (K) cannot be useless, or even harmful and with a negative value relatively to other goals of us*. Value is always in relation to several goals and dimensions; and that Inf Item (although true) can be ambivalent: good as for the goal of truth but bad as for other goals or functions.

It seems to me that this view is not contradictory with a pragmatic definition of truth; the item could remain true even conducing us to some failure on some goal. It is successful, valid, for certain actions but not for others or for additional goals it elicits or for bad inferences it implies.

Let’s say something not in relation to our (rather preliminary) view of “knowledge” in strict sense, and of truthfulness, but in relation to the classical view of truth: the assumption correspond to the ‘real’, objective (subject independent) state on the world. Like in logical definition of the operator “to Know”, where (Know X P) = (Believes X P) & P. “X *knows* P” means that “X believes that P” and “P is true” (from the perspective of an ‘observer’ assumed as the real state of the world).

In this classical (and common sense) view of truth the questions are:

- Is a True Inf (a “knowledge” in strict sense) necessarily and always good, useful, valuable?
- Can be a “false” belief/assumption useful, effective, for a given goal achievement, not accidentally but thanks to its falsity or partiality (ignorance)?

As for (A) the answer is: It depends! “Good” in relation to which *set* of goals, to which evaluative *dimensions*? As we said, for the goal of known the truth, that K Item has always value, it contributes to; but relative to other goals it is pertinent and relevant for, it can be useless and even negative, harmful; although ‘true’.

Let us just focus on some specific *negative effects of “true” Ks* (putting aside the fact that they can produce pain and X can even prefer to die that to live with such a painful truth!):

- True but useless, irrelevant Ks; so I wasted my time or money or (mental or practical) efforts, and perhaps I have lost other opportunities.
- True but harmful since they activate (affective or automatic) reactions hindering or counterproductive for me; for example, they trigger panic and this creates obstacles for a more successful behavior. Or I know of a plane crash and I decide do not take my fly. Or harmful since to posses that Inf expose me to be tortured to extort it or to be killed.
- True but noxious since they are cognitively deviating, by activating biases and not so rational decisions; for example, ‘monetary illusion’ bias and a seeming wage increasing.^42^ Or like in the celebrated experiment of Wertheimer where a geometry problem was better solved by people ignorant in geometry, since people studying geometry read it in a prejudicial framework.

As for (B), let’s add that:

- To arrive to know the truth, to have a more realistic and truthful view of things isn’t always an advantage for cognitive agents. It can *reduce* the possibility of success of our goals. That also because our ‘expectations’— that we formulate on the basis of our beliefs—can have effect on the results and be ‘self-fulfilling prophecies’. It is a well-known result of psychological research that ‘optimists’ (sometime a bit gullible) systematically distort (in their favor) the perceived probability of the event and their control over; while ‘pessimists’ have a more realistic and rational view of that. Nevertheless, this attitude of optimists favors them and supports the *success* of their expectations. How is it possible that a partially deviating cognitive representation of the word be an advantage? The reason is that we play two very different kinds of “lotteries” in our life. Those were the probability of a given result is a priori determined and not influenced by the player; like to gamble with dice. It is useless that I “feel sure that…”, that if I play with my left hand the result will be the desired one, and so on. The probability is given (in this kind of games pessimists should go better). However, there are other ‘lotteries’ in life (like courtship, like a negotiation, or a job interview, an exam,..) where my expectation, hope and trust can make the difference. If I’m quite pessimistic I will invest less, I will persist less and be easily discouraged, and I also will present a worst ‘image’ of me and my self-confidence. However, the result of this ‘lottery’ is changed by how much I invest, and persist, and from my self-confidence message to the others. In other terms, my attitude affects the probability of my success.

Thus, a partially *unrealistic and incorrect Inf and beliefs can help*.

More in general, also at the social-political level there is a utility of illusory^43^ and utopian beliefs. They play a parabolic role for human ambitions and objectives: in order to rich X we have to aim to in Y.

*“*By striving to do the impossible, man has always achieved what is possible. Those who have cautiously done no more than they believed possible have never taken a single step forward.*” Michail Bakunin*

## The utility of K or of believing K?

That a given Inf, a given D/K has a Value, and more precisely an Utility (in relation to our Gs and uses), does it mean that we believe in it “*because* this is convenient” for us? In other words: can we reformulate the problem of the Utility of K (that P) as “convenience to believe’? “It is convenient to me to believe it, then I believe it!” No. We have not to confuse or identify the advantage, utility of K (if believed and used)^44^ with the advantages, utility of believing that P.

### Deciding to believe?

First of all, the basic mechanism/process of ‘deciding’ to believe or better to ‘arriving’ to believe is not a true ‘decision’, based on advantages and convenience; I like to believe that P so I decide to believe it. This is in case just a deceptive and unconscious process of ‘motivated reasoning’, or a ‘defensive mechanism’ (Miceli and Castelfranchi 1998). We cannot for example believe that something is true just because X pays us if we believe so, or because X would harm us if we do not believe so. ‘Economic’, rational, convenience doesn’t govern the believing ‘decision’; we can just declare to X and simulate that we believe what he likes. Believing process has its own bases: source credibility, supports, evidences (section “The value of knowledge and its origin”); it is an epistemic process not a utility driven one. And the convenience of a given belief cannot (consciously) alter its ‘*credibility’.*

Promises, threats, rewards, are useless for inducing to believe. This is one of the guaranties of our ‘autonomy’ and defense from social manipulation.^45^ Thus, we do not believe something (D/K) *because* it is convenient for us to believe it. However, to believe it is convenient and makes D/K convenient, useful (or dangerous). Moreover, as we said, the degree of certainty, how much we believe in D/K, increases the subjective value we ascribe to it. We feel safer while betting on it, and we preserve it strongly.

Let’s assume that P is good, useful for X; then also knowing (believing) P is useful for X. If a given K has value for me and is objectively useful for me, it is convenient to me know it and believe it; but I do not believe it because I think that it is convenient to me believe it: I believe that it is probably true, reasonably grounded, and useful for my goals.

In sum, we might even reformulate the problem of K Utility as ‘convenience to believe/assume’, provided that it remain clear that:

- Utility is the usability and efficacy of K for the subject goals, and its certainty/safety;
- To believe or not to believe K does not become an economic ‘decision’, based on the utility to believe.

## Social dimensions of knowledge value

We will not focus on the crucial social dimensions of the theory of K Value; like:

- K as a *competitive resource* and the advantage of its unique possess;
- K as a Common (Hess and Ostrom 2006); “reciprocal altruism of K” (Conte and Castelfranchi 1995);
- K exchange and circulation;
- Deception and its utility;
- Why K is ‘power’ in social sense (Bacon’s citation); Dependence etc.

Let’s us just give two nice examples of the Value of K due to its social functions.

### The value of information for our image asset

A really special value acquire Inf Items about us and our behavior and this is why we want the other know them; or—vice versa—the relevance of the ‘secretes’ about us, what we hidden and don’t want the other know. The function of this maneuver and the positive/negative value of that Inf Items is the shaping of our social ‘image’ and of evaluation about us, then of our esteem, trust, reputation,..

Our comparative evaluation (due to the good information about us and the hidden of the bad ones) is also vital for us because it is the substance of human social (non formal) hierarchies, and of our positioning in them (prestige, visibility, and relational capital, opportunities..). We socially live of what the other know and believe about us.

### To share or not to share K? Effects on its value

A general question is:

When and why that a K/Inf be shared gives it an additional and special Value?

When and why the circulation and sharing of K reduces its value?

When K has value only if it is personal, private, or of restricted group?

Let’s give two examples of possible reasons for K sharing:

*A) Common ground and membership* As we said, a crucial criterion of *certainty*, credibility is *the number of convergent sources*. So, notice that the fact that other people believe P or say that P increases the probability that me too believe so. Also for two other crucial social reasons:

- *In order to interact and communicate* whit other people (as we need) and to reduce uncertainty in coordination, cognitive activity, … we need a ‘common ground’; that is, to share a lot of K, and *to give for granted that we know as they know* (and vice versa), and rely on that.

So this is a *value* and crucial functional utility of sharing K.

- Moreover, sharing K, believing as and what the other believes is *a condition for being and feeling “part of them”, a member* of a given group, community, culture. Since we need to be part of, to belong, this is an additional function of sharing, constructing together, K.

*B) Intimacy: K to be shared and not to be shared* A very strange/remarkable form of shared/not shared K, with its ‘rules’ in order to be and work *‘as’* “intimacy”, are those Ks about our body, our thinking and feelings, our story and narrative, that not everybody has to have access to (to see, to listen to,..). *Otherwise I would not have ‘intimacy relations’ and ‘intimacy’ parts of me*.

So, the rule is that there are *people that has****not****to know* (see,..) those Inf about “me”; I have to ‘protect’ and hidden them (decency,..); while there are *other people (my intimate relations) that****has to****known;* I have to share these K with them, otherwise they are not really in ‘intimacy’ relation with me.

And this adds, gives special (social) Value to those Ks.

## Concluding remarks

A theory of the Value/Utility of information and knowledge (K) is not really there. This would require a theory of the centrality of Goals in minds (for motivating and regulating adaptive action), and of the role that K relative to Goals and their dynamics, management.

K Value is a notion relative to Goal Value. Inf/K is precisely a resource, a means and the value of means depends on the value of their possible functions and uses. We search for, acquire, buy, preserve, use, consume,.. exchange… this crucial ‘power’ for achieving goals.

‘Relevance theory’, Information theory, Epistemic Utility theory, etc. are not enough for providing a theory of the Value/Utility of K. And also truthfulness is not ‘the’ Value of K.

K has also costs and implies risks; it can not only be useful but negative and dangerous. In a sense, one should apply to K – in this goal-oriented perspective – an ‘economic’ frame.

Moreover, Goals give value to K, since K *serves* for G achievement (and also planning, choice, decision..), but also the other way around. It is a *dialectic relation*: K gives Value to Goals since their value depends on beliefs about their outcome (expectations), about consequences (harms, costs,..), their probability, etc. And also – for the ‘felt’ not ‘reasoned’ Goal Value—from beliefs about possible pleasant/unpleasant events, the memory traces of emotional experiences or sensations (somatic markers): this information/K items gives value to that possible Goal.

## Appendix 1

Also this issue is of course much more complex and subtle. For example, it is even possible that a given representation “says” something about something Obj’ that is not in its content! It can (be used for) inform us that Obj’ is absent, is not there, isn’t the object of that representation or predicate. However, since the set of absent content is infinite, in order that representation being “about” Obj, in fact it is necessary another representation that explicitly contains Obj’. Either:

i. From Repp’ (where Obj is NOT there) we can infer a Repp” strictly and directly about Obj. For example, from R’: “Mary loves Paul” (& “Mary loves only one man”) we derives R” that “Mary does not love John”. Thus R’ in *indirectly* about John; but it is necessary that the R’ where John is not contained is combined with another R where it is there. Or:
ii. An image where there is no Obj’ (a given cat), informs us that “the cat was/is not there”, but only if we put this R’ in relation, compared with, another representation R” containing (directly about) that cat and we—by comparing—see that that Obj’ (and not un infinite number of other Objs) is NOT there.

## Appendix 2

Another bad mess, typical of psychological approaches to ‘motivation’ (that should actually be called “the theory of goals and their origins and dynamics”) is *the (implicit or explicit) identification of “motivation” and “emotion”*. It is true that emotions are one of the sources and triggers of goals: this is part of their “conative” nature; these are “impulses”, and part of “drives”. And it is also true that emotions are “signals” of the status of our relevant goals; they monitor some important and specific goals of ours (fear is about safety; shame is about social image; pride is about our value, mastery and sense of competence; etc.). In other words: a theory of emotions intrinsically requires a theory of goals: *no emotions without goals.* But *not* the other way around. The theory of goals is *not* grounded on emotions and does not intrinsically require them. We can have a perfect ‘goal-directed’, intentional, agent without any emotion. What is intrinsically needed is a ‘value’ of its goals/motives; either due to selection or to learning or reasoning.

## Footnotes

1 Let’s simplify this broad category and set of mental representations (including ‘beliefs’, candidate beliefs and data, assumptions, beliefs grounded on reliable evidences and sources, beliefs that the system meta-believe to be ‘true’ and uses as such, expectations, hypotheses,…) by using the term “knowledge” (as rather traditional in AI and Cognitive Science models) for all the doxastic representations that the Agent builds and employs, for relying on them when acting or deciding. I will also use the more neutral terms of 'Information item' or 'data'. I will discuss later the problem of Information items value in relation to ‘true knowledge’ and the value of the truth.

2 The cited Bacon's sentence does not refer only to social power, to the fact that K gives *power over the others* since makes them dependent on us; but more basically it refer to the fact that to know means to be able and in condition of, to have the 'power of' realizing something.

3 For example: “System Oriented Theories do not try to define but presuppose, the fundamental concept of relevance understood as a relation between some information and an informee”…. “The concept of relevance is arguably the fundamental concept of IR [information retrieval]. […] we purposely avoid giving a formal definition of relevance. The reason behind our decision is that the notion of relevance has never been defined precisely in IR. Although there has been a large number of attempts towards a definition of the concept of relevance, there has never been agreement about a unique and precise definition”. Crestani et al. (1998) (cited by Floridi 2007).

4 In their frame what we find definitely correct is: the context dependent view of Relevance; its comparative and not absolute character; the change of the salience; the idea of the costs and "efforts" (although just reduced to cognitive "processing effort"); the idea of human cognitive tendency: “Relevance theory claims that humans do have an automatic tendency to maximise relevance, not because we have a choice in the matter—we rarely do – but because of the way our cognitive systems have evolved. *Cognitive Principle of Relevance*: Human cognition tends to be geared to the maximisation of relevance”.

5 We will also contest that only true beliefs are useful/positive, and false ones are always noxious.

6 In our view the notion of ‘Relevance’ has also been mixed up and has obscured another crucial notion and theory: that of ‘Pertinence’: what is ‘about’ what I’m interested in (see section “Where is ‘pertinence’ theory?”, and Note 14).

7 I will in particular exploit for synthesis a very clear contribution of Floridi “Relevant Information or How philosophy can help you find the information that you really need.” 2007. For a much more extended and deep theory see (Floridi 2011).

8 “Interest” is a precious term, but ambiguous: interest just as additional information, for epistemic finality (“*epistemic utility”)*, for curiosity or understanding something; or for practical advantages, for my ‘interest’ = ‘convenience’? (see section “Other distinctions”).

9 As for this never defined crucial notion see section “Where is ‘pertinence’ theory?”.

10 See section “The epistemic integration value of K items” and (Paglieri and Castelfranchi 2005) for our less formal/strong but more psychological approach to this problem.

11 In a quite ‘normative’ and prescriptive sense: how we *should* correctly believe.

12 I have to thank Emiliano Lorini for signaling to me this literature. See also Howard (1966), with the notion of the expected value of perfect information or of sample information. In the same perspective it is taylored the definition of "Value of Information" in the Business Dictionary: "Maximum price one should pay for knowing the actual value of an uncertainty before deciding on a course of action". http://www.businessdictionary.com.

13 For example, distinguishing when the Inf produces an improvement of the probability to achieve the goal or the value of the goal or the degree of its achievement.

14 In French texts actually Sperber and Wilson use the term “pertinance” for “relevance”. This is the French meaning of this word, but not the English or the Italian one (more related to the Latin original meaning). “Pertinent” means that “pertains”, while “relevant” means “important”, “considerable“.

15 For a more systematic theory see Castelfranchi (2012).

16 Unfortunately in English the word "goal" tends to have such restricted connotation.

17 Although in fact any possible action actually (consciously or unconsciously) is an “attempt” and relies on some external process/agent for its accomplishment (Lorini et al. 2006).

18 For the broader theory of ‘desires’ see Castelfranchi (2012).

19 Every goal failure is a 'frustration' and any attainment is 'satisfaction', but not necessarily with a significant and felt subjective experience of pain, irritation and disappointment in the first case, or of joy or pleasure in the second one.

20 For a general theory of “needs” (“having an objective need” vs. “subjective needs” vs. “felt needs”) see Castelfranchi (1998).

21 Notice that (differently from “desires”) “needs” intrinsically are “instrumental” goals. I need something *for* something; it is necessary *for* realizing something. Either for achieving a practical goal (practical need: I need the key for opening the door), or for stopping or preventing a sufferance.

22 Of course, this value is not the only factor which determines the decision to do or not to do something, or the choice between two competing goals. The goals’ degree of “urgency”, the estimated probability of achieving them, the involved costs, the perceived risks and possible harms are factors which also come into play.

23 This is why we need a given value of the top-level active goals, or final motivations: at a given moment, given our age, gender, personality, culture, personal biography, identity and tastes, we have certain final motives with a given value for us, and we can choose between different active goals; and/or we have certain active drives or felt needs and desires, but also our long term objectives with their subjective terminal value.

24 Slovic’s idea of “affect heuristics” just partially captures this aspect and solves the problem of its modeling (Slovic et al. 2002).

25 Remember Seneca’s sentence: “We are so unhappy as we believe to be”!.

26 'Standard' is a 'Quality' (the feature that makes Obj good for, apt to, in condition of, with the power of,..) generalized to its class or kind: which properties/features has to have this *kind* of Obj in order to be good (for..)? After having identified the Qualities and generalized the Standards the Evaluation process of Obj (if Obj is good or not for) changes it nature. We no longer need to test Obj, in order to see if it produces the desired effect (goal); it is enough to see whether it has the needed qualities; or if it correspond to the standard. We know if something is good or bad without direct experience of it; as an inferential generalization and instantiation: since what make an Obj good-for G is Q1 and Q2, and since Obj' has such qualities, then it is goof for. We have qualities and standards also for information and K acquisition. We know that a given Inf/K is good also without and before 'testing', using it.

27 The greater the perceived V, the preciousness of a given information, the greater the Value of the possible (sub-)goal of acquiring and using it.

28 Psychologically the problem might be more complex; since one might perceive the contribution of several - although necessary - data as not equivalent for the goal achievement. How much G achievement depends on that D more than on the others?

29 The *context-dependent nature of KV* is of course more general (since also Goals and their value are context-dependent). An example of the context-dependent ‘value’ of a given K is a question during an examination or in a TV contest: “When is Garibaldi born?” If you know that, if you answer correctly, you win 10.000$! But after that moment, that K has no longer this ‘value’!

30 For example, I want to be rich, and I can become the richest person in world, or very rich, or riche, or quite rich,..

31 That is, from the perspective of an ideal ‘observer’, assumed as perfectly informed and rational.

32 Truth is a thing of this world: it is produced only by virtue of multiple forms of constraint. And it induces regular effects of power. Each society has its regime of truth, its “general politics” of truth: that is, the types of discourse which it accepts and makes function as true; the mechanisms and instances which enable one to distinguish true and false statements, the means by which each is sanctioned; the techniques and procedures accorded value in the acquisition of truth; the status of those who are charged with saying *what counts as* true' (Foucault, in Rabinow 1991).

33 This is a bit more complex since in certain cases the ‘source’ (like in communication) can also inform about its own degree of certainty: I trust very much that source but she says “I guess that P, but I'm not fully sure”; while another source that I do not trust so much might say “I’m absolutely sure that Q”. How to combine and weigh up the certainty evaluation from the source with the reliability of it?

34 Again: we use ‘belief’ as prototype of doxastic representation of many kinds; this applies also to ‘true’ knowledge items, to mere ‘assumptions’ (I’m not sure that P, but decide to proceed “as if” P was true), to hypothesis, to predictions, opinions, etc.

35 For a similar – but less analytical view, with a rather limited theory and ontology of goals – see also the well-known “theory of Reasoned Action” by Fishbein and Ajzen (1975) and also Kruglanski (1996).

36 On the cognitive processes and utility of defense mechanisms like ‘denial’ see (Miceli and Castelfranchi 1998); we defend us from K, ideas, mental representations!.

37 The notion of "costs" is ambiguous; it concernes what we have to spend, sacrify, in order to acquire/achieve something, but also bad consequences, effects, risks of having or not having something. This ambiguity gives rise to this nice paradox of Derek Bok: "If you think education is expensive, try ignorance." Not having a relevant K can be very noxious and "expensive", however also having K can harm us (section “Useless and harmful Inf”).

38 However consider that sometimes (self)deception (for example by 'denial') is useful for the subject.

39 A possible definition is: I believe P and also believe that I believe P on a valid base, evidence, and that P correspond to the external, independent, 'real' world, and would match if I would controll/test its correspondence by an epistemic action.

40 This 'evidence' can also be wrong; the action can be successful although the assumption was actually wrong ('objectively', that is, from the observer's point of view).

41 But not the goal to know everything that is true in the world.

42 One might say that this is due to the incompleteness of that Inf or not fully understanding. However, on the one side, K is always incomplete; on the other side, here we are evaluating the value of a given K item. A K can be useful joined with other Ks but can be deviating if alone or with other wrong Ks. It can be “true” but bring to wrong or noxious conclusions.

43 To saying nothing about Leopardi’s thesis about the need for ‘illusion’ in human life and sufferance.

44 Of course in order to be ‘used’ and thus useful in strict sense a D must be ‘believed’ or at least ‘assumed’ “as if” believed. If I do not believe it, reject it, it is not directly useful (has no Value for me), except the utility that precisely rejecting it (and not doing something, believing something else thanks also to it as rejection, or defending other Ks) might have.

45 Notice that what in this situation matters for Y and has value (for example what X 'buys') is not P, the content of the belief, but believing, the status of accepting to believe. Often it is even irrelevant the real content P, in order to show appeasement. What is useful to Y (and what X expects) is not P, but "Believes Y P"; or more precisely that "Believes X (Believes YP)".
